# A Content Analysis of Media Coverage of the Introduction of a Smoke-Free Bylaw in Vancouver Parks and Beaches

**DOI:** 10.3390/ijerph10094444

**Published:** 2013-09-18

**Authors:** Arezu Moshrefzadeh, Wendy Rice, Ann Pederson, Chizimuzo T. C. Okoli

**Affiliations:** 1 British Columbia Centre of Excellence for Women’s Health, E311, 4500 Oak Street, Box 48, Vancouver, BC, V6H 3N1, Canada; E-Mails: wendymrice@gmail.com (W.R.); apederson@cw.bc.ca (A.P.); 2 Experimental Medicine Program, Department of Medicine, University of British Columbia, Room 10226, 2775 Laurel Street, Vancouver, BC, V5Z 1M9, Canada; 3 College of Nursing, University of Kentucky, 315 College of Nursing Building, Lexington, KY 40536, USA; E-Mail: ctokol1@uky.edu

**Keywords:** smoke-free policy, equity, content analysis, media

## Abstract

The Board of Parks and Recreation in Vancouver, BC approved a smoke-free bylaw in the city’s parks, beaches and recreational facilities, effective 1 September 2010. We analyzed local news coverage and portrayal of the bylaw to understand the potential influence of news media on public perception of the bylaw in order to inform the media advocacy work of public health interest groups. We compiled a data set of newspaper articles (n = 90) and conducted a quantitative content analysis to examine content related to the outdoor smoke-free policy, including article slant, topics related to smoking and tobacco control, and any equity-related concerns raised. Newspaper coverage in Vancouver was largely supportive of the outdoor smoke-free bylaw. However, concerns over rights were frequently discussed in letters to the editor. Such equity concerns were rarely discussed in news articles, showing a potential disconnect between the concerns expressed in the media by members of the public and the coverage provided by print media.

## 1. Introduction

In April 2010, the Vancouver Board of Parks and Recreation approved a bylaw completely banning smoking in the city’s parks and on the beaches. The bylaw was initiated by Vancouver Parks Board staff in an effort to reduce secondhand smoke exposure, eliminate potential environmental problems (such as fire risk and litter) and ensure smoke-free role-modeling for children and young people [1]. 

There was some consultation with the public during the development of the bylaw, most notably a public web-based survey conducted through the Board of Parks and Recreation website during October 2009. The general public was also able to provide feedback regarding the bylaw to the Park Board Commissioners at the April 2010 Board Meeting at which the bylaw was debated. Following consultation with the public, the Park Board members voted unanimously in favour of the bylaw. After the necessary amendments to the existing health code were made by the City Council, the implementation date was set for autumn 2010. The Park Board spent the intervening months focused on educating the public about the impending bylaw. They actively worked with local media to raise awareness in the months leading up to the formal implementation of the bylaw on 1 September 2010 [1].

From the perspective of the Parks Board and City of Vancouver, raising public awareness of the bylaw was particularly important in ensuring that the bylaw was successful in its primary aim of reducing smoking and secondhand smoke exposure in outdoor public recreation areas [2]. The city of Vancouver has over 200 parks and ten ocean-side beaches; given the large area of outdoor space that would be affected by the bylaw and the relatively limited number of Park Ranger staff—who were designated as key enforcers of the bylaw—compliance with the bylaw was anticipated to be a challenge [1]. Park Board staff and Board Members therefore envisioned that with sufficient public awareness and acceptance, the bylaw would be essentially self-enforcing [1]. 

Given the important role of news media in the dissemination and impact of health-related issues and policies [3,4,5], we examined print news reports concerning the development and implementation of the outdoor smoke-free bylaw in Vancouver. Our objective was to assess media coverage and content both pre- and post-bylaw implementation in order to understand the role local print media may have played in the public’s perception of the bylaw through generating a description of the media’s coverage of the bylaw. The majority of existing media analyses have only examined indoor smoke-free regulations or tobacco control regulations in general [4,6,7,8,9], without specifically addressing the issues of outdoor smoke-free policies. We were particularly interested in the coverage of issues that were unique to the regulation of public spaces, namely equity concerns. These concerns included the rights of smokers and non-smokers, fairness, and effects on disproportionately affected population groups.

## 2. Media and Public Opinion

News media are an important source of information for the general public regarding health and health policy news [3,4,5]. Previous studies have confirmed the strong agenda setting effects of print media. While news media do not necessarily tell the public what to think about particular issues, they are remarkably successful at telling the public what issues to think about [3,10,11,12]. By covering an issue, the media increases the relative importance of that issue [8], and by reporting on some issues and not others, the media influences what issues people think about [7]. The news media acts as a conduit for information between policy-makers and the general public, and ‘provides cues about what issues should be on the forefront of people’s concerns’ [3]. Hence, print media, as a major information source for many citizens, particularly in relation to local issues [7], ‘can shape public opinion and expectations about policies that, in turn, influence policy development processes’ [5]. 

Local news media have a potentially influential role to play as community resources in communicating local health policies [7]. Print media, in particular, play a valuable role in the adoption of tobacco control policies, with Menashe and Siegel [9] finding that tobacco-related news coverage helped determine people’s thoughts on tobacco and guided how they approached tobacco use as a social issue. Indeed, Clegg Smith *et al*. [4] found that print media had a stronger relationship to municipal tobacco bylaw uptake than either scientific research dissemination or political discourse. In an event history analysis of municipal smoking bylaws in Canada, Asbridge [6] also found that the print media and health advocacy play the strongest role in explaining the adoption of local-level smoking bylaws.

## 3. Methods

We conducted a quantitative content analysis of print news media to examine content related to the smoke-free bylaw in Vancouver such as article slant, appearance of topics and themes related to smoking and tobacco control, and equity concerns. Four popular local and provincial newspapers were selected and articles were obtained using the Canadian Newsstand Database as well as independent newspaper archives for newspapers not included in the database. Articles from 1 January 2010 to 31 December 2011 were included in order to examine both the period prior to the announcement of the bylaw and the period following its announcement and implementation. The following search terms were used to identify articles pertaining to the bylaw:

- smoking, or tobacco, or smokefree or smokers or smoke-free, and,
- ban*, or bylaw, or control or regulation* or restrict*, and
- park*, or beach*, or outdoor*.

Articles were excluded from our study if the depth of discussion regarding smoking or tobacco use was limited to the mention of the search term only (e.g., mention of tobacco-stained teeth in an article otherwise unrelated to smoking/tobacco), or if the search terms were used in a manner unrelated to smoking/tobacco use (e.g., colloquial use of the word “smoking” to mean “attractive”). After 68 articles were excluded, the final dataset was comprised of 90 articles.

Coding of the articles was performed in two phases. The first phase involved using a Perl script that was designed for this specific set of articles. This script scanned the articles and identified the newspaper name, article date, author, word count and page number, which was then automatically entered into a spreadsheet from which a random sample was checked by a coder for accuracy. As these articles’ details were consistent across all the articles and did not require interpretation, it was thought that automating this process would minimize human error. In the second stage, articles were coded using a Media Framing Codebook adapted from Clegg Smith [13] and *An Intervention for Promoting Smoke-free Policy in Rural Kentucky* [14]. The codebook consisted primarily of categorical variables in order to reduce the possibility of coder subjectivity and to allow for both a descriptive and quantitative approach to the data analysis. 

The codebook contained approximately 45 content variables related to smoking and smoke-free regulations in six categories:

1. Article Relevance: an indication of the relevance of the article content towards smoking and/or smoking regulation (smoking focus/non-smoking focus)
2. Geographic Focus: the geographical focus of the story (local, provincial, national or international)
3. Slant: the article’s slant towards smoke-free policy (positive for smoke-free regulation, neutral towards smoke-free regulation, negative towards smoke-free regulation or n/a)
4. Primary Approach: the primary approach taken to tell the story (Social, Environmental, Health, Rights, Factual, Regulation or Other)
5. Theme: the overall theme of the article that made it a news story for that day
6. Topics: coding the mention of numerous topics related to smoking/tobacco control (such as financial issues, health information and equity issues).

Coding was conducted by one team member who was blinded to all identifying information except for a randomly assigned article identification number. A second coder coded a random selection of 20% of the articles to assess inter-coder reliability. Using Cohen’s kappa, there was a mean score of k = 0.768 with scores ranging from k = 0.643 to 0.913 showing substantial to almost perfect agreement [15], indicating a high level of inter‐coder reliability.

## 4. Results

A total of 90 articles were coded, of which 82.2% (n = 74) had a smoking-related focus. The majority of articles were focused on Vancouver (61.1%, n = 55), with the remaining articles concentrated on international news (11.1%, n = 10), unspecified geographic areas (7.8%, n = 7), provincial news from British Columbia (6.7%, n = 6), other localities within the province (5.6%, n = 5), other provinces within Canada (4.4%, n = 4) and a national focus (3.3%, n = 3). Almost two-thirds of the articles were news stories (60%, n = 54), 18.9% were letters to the editor (n = 17) and the remainder were columns, opinion pieces and editorials (n = 19).

**Figure 1:**
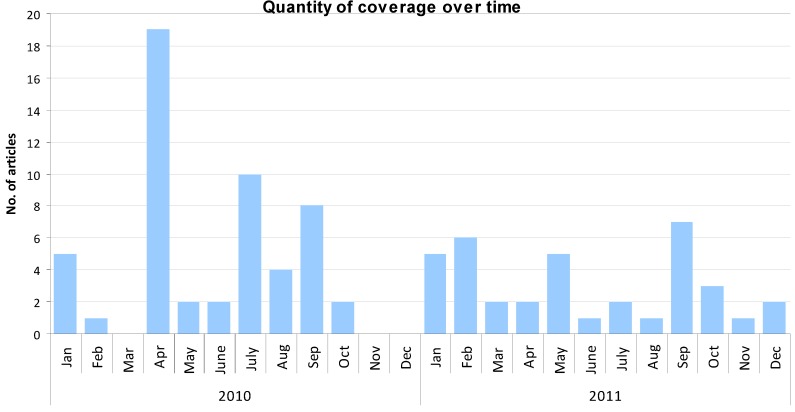
Quantity of coverage over time.

The article publication dates ranged from 19 January 2010 to 27 December 2011, as illustrated in Figure 1. Peak coverage of the bylaw occurred in April 2010, when 19 articles were identified, likely due to the public announcement of the approval of the bylaw on 20 April of that year. This month also had a greater number of letters to the editor than other months (n = 9). To a lesser extent, coverage rose in July 2010 (n = 10), primarily due to a temporary smoking ban implemented at that time due to fire risks. The number of articles showed a slight increase in September 2010 (n = 8) when the bylaw was implemented, and again in September 2011 (n = 8) with the announcement of a similar smoke-free policy across the Greater Vancouver Regional District.

### 4.1. Article Slant

The slant of each article was examined showing that 38.9% (n = 35) of the coverage was positive towards the bylaw, 30.0% (n = 27) was neutral, 22.2% (n = 20) was negative towards the bylaw, while the slant could not be determined in the remaining 8.9% (n = 8) as there was not enough content related to smoke-free policy in the article. The article slant was then analyzed for the two most prominent article types: news articles (n = 54) and letters (n = 17) (see Table 1). Among news articles, half held a positive slant towards the smoke-free policy and only 7.4% of the articles were negative towards the bylaw. Comparatively, letters were mainly opposed to the bylaw, with 64.7% of articles having a negative slant.

**Table 1:** Article slant.

	Article Slant			
	Positive (%, n)	Neutral (%, n)	Negative (%, n)	N/A (%, n)
**All Articles**	38.9%, 35	30.0%, 27	22.2%, 20	8.9%, 8
**News reports**	50.0%, 27	38.9%, 21	7.4%, 4	3.7%, 2
**Letters to the Editor**	23.5%, 4	5.9%, 1	64.7%, 11	5.9%, 1

**Figure 2:**
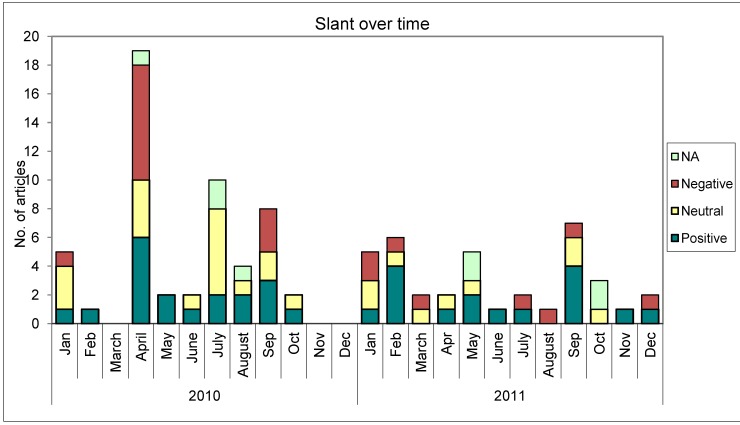
Slant over time.

When examining slant over time (Figure 2), articles with a positive slant were spread throughout the two-year study timeframe, while articles with a negative slant were more concentrated in April 2010 when the bylaw was announced, and were not found in every month.

### 4.2. Article Approach

The most frequent approach used was a health approach (38.9%, n = 35) which encompassed articles emphasizing various health-related issues in relation to smoking and/or the bylaw. Articles that were simple factual accounts, and articles with an environmental approach (emphasizing the environmental implications of smoking and/or the bylaw) each accounted for 18.9% of all articles (n = 17 for each approach). The remaining articles used either a social approach emphasizing the social issues relating to smoking (such as nuisance and social modeling, n = 11), a rights approach raising issues regarding rights in relation to smoking and/or bans (such as the right to smoke and the right not to be exposed to smoke, n = 11) and a regulation approach emphasizing issues related to the creation of bylaws and/or regulation of laws (such as overregulation or the need for more social laws, n = 10).

Articles that were news reports primarily used a health approach (n = 32) followed by a factual approach (n = 14) and environmental approach (n = 13). In contrast, letters to the editor most frequently used a rights approach (n = 5), followed by a regulation approach (n = 4).

In analysis of the relationship between article approach and slant, we found the following principal slant for each article approach:

- Health—**Positive **(65.7%, n = 23)
- Environmental—**Neutral **(52.9%, n = 9)
- Factual—**Neutral **(58.8%, n = 10)
- Rights—**Negative **(81.8%, n = 9)
- Social—**Positive **(63.6%, n = 7)
- Regulation—**Negative **(60.0%, n = 6)

Regulation and rights approaches contained more negativity towards the bylaw, while support for the bylaw was strongest in articles with a health approach.

### 4.3. Topics

A range of specific smoking/tobacco control topics were coded if there was any mention of the topic in the article. These topics were grouped into six major topics, as outlined in Table 2. The most frequently mentioned topic was that of enforcement and implementation (mentioned in 64 articles). This group of articles included those covering issues such as signage regarding the smoke-free bylaw, Park Rangers and police officers as enforcement agents and general implementation issues. The second most frequent topic was unintended consequences of smoking, mentioned in 39 articles, which primarily covered mentions of litter, fire and public nuisance. The topics mentioned least frequently were financial issues (mentioned in 14 articles) and equity issues (mentioned in 21 articles). Financial issues covered topics such as the societal and environmental costs of smoking, cigarette taxes, costs of bylaw enforcement and healthcare costs of smoking and second hand smoke. Equity issues were topics concerned with rights of smokers and non-smokers, fairness and groups disproportionately affected by the bylaw.

**Table 2:** Article Major Topics.

Topic	Number of Articles
Enforcement and Implementation	64
Unintended Consequences of Smoking	39
Second Hand Smoke	31
Negative Health effects to Smoker	28
Equity Issues	21
Financial Issues	14

## 5. Discussion

Over the time period examined, there was generally low coverage of the smoke-free bylaw, with only 90 articles over a two year period. This was most likely due to competing news stories, and particularly the news coverage of British Columbia’s harmonized sales tax (HST). The debate surrounding the introduction of the HST, as well as the crusade-like efforts to petition against it, dominated news coverage in the summer months of 2010, and again during the HST referendum over the summer of 2011. This likely impacted the coverage of the smoke-free bylaw, which would have been most relevant to the public over the summer months as well.

Overall, Vancouver’s new outdoor smoke-free bylaw was presented with a positive slant in the news media. Notably, the voice of the public portrayed in the news media, represented by letters to the editor, was primarily negative toward the bylaw; however, as there were fewer letters than news articles, readers were left with an overall generally supportive slant towards the smoke-free bylaw. 

General coverage of the smoke-free policy focused on health reasons for introducing the ban. This focus increases the potential for the public to view health as the salient issue regarding the new smoking regulation. News articles—which made up over half the sample—identified health and environmental factors as the primary reasons for supporting the smoke-free policy. Letters to the editor, however, had largely different concerns, namely, issues of individual rights and over-regulation of public spaces. While these concerns were overwhelmingly raised in letters, they were rarely addressed in news articles themselves, showing a potential disconnect between the concerns of some members of the public and coverage of the issues surrounding the bylaw by news media.

Support for the smoke-free bylaw was largely centered on health concerns as a justification for the bylaw, as articles with a positive slant focused primarily on a health approach. However, health-related topics themselves were not frequently mentioned in the articles, showing a lack of presentation of health information. Thus health concerns were used as validation for the need for the bylaw despite a lack of presentation of any scientific evidence of health impact of smoking in outdoor public spaces. This lack of presentation of evidence in the media to support health statements is consistent with previous findings regarding other Canadian health initiatives [16] and should be highlighted as an area to be further examined by media advocacy groups.

By a large margin, the topic addressed most frequently in the news was that of enforcement and implementation of the bylaw. Financial issues and concerns regarding equity were infrequently mentioned, and as such, could be regarded as less important issues by readers. The low presence of equity-related issues could be of particular concern because the smoke-free bylaw in Vancouver affects *public* spaces. As such, discussion of concerns unique to regulating individual behaviour within a public environment is important in order to understand the context in which the bylaw is implemented and its potential impact on the community. This discussion was clearly lacking in the media coverage of the bylaw, despite an overwhelming indication in letters to the editor that these issues were a topic of public concern.

Coverage of Vancouver’s smoke-free policy was greatest in the month that the ban was announced, and to a lesser extent, in the weeks prior to its implementation. In the month with the greatest quantity of coverage (April 2010), however, articles that were negative towards the bylaw were more frequent than coverage supporting the bylaw. It would appear that the voice of the public as presented in the media through letters outnumbered the voice of the news media itself at that time. In short, for a brief period, the media were a platform for the voice of the public rather than a source of news and information regarding the smoke-free bylaw.

Previous research has highlighted the relationship between the quantity of media coverage of an issue and the subsequent importance the public then attributes to that particular issue [5,17,18]. These findings suggest that the potential for the Vancouver-area news-reading public to have perceived the smoke-free bylaw as an issue of importance would have been the highest at the time of the announcement of the bylaw due to the increased coverage. As such, the month immediately following the announcement of the bylaw would likely have been the most favourable time to affect public opinion regarding the policy through the news media. 

## 6. Conclusions

Local print media’s coverage of Vancouver’s smoke-free bylaw had a mainly positive slant, particularly in news stories. Coverage focused on health reasons for the ban, increasing the potential for the public to place importance on health as the salient issue regarding smoking regulation. There was a lack of coverage of equity issues despite the concerns expressed in letters to the editor as well as the relevance of these issues to the regulation of public spaces. The potential for agenda setting effects was greatest when the concept of the ban was introduced to the public. Health advocates, human rights advocates and other groups with a vested interest in bylaws such as this may wish to use the time immediately following the announcement of the bylaw to inform the public of their respective platforms.
